# Retardation effect of nitrogen compounds and condensed aromatics on shale oil catalytic cracking processing and their characterization

**DOI:** 10.1007/s13203-015-0131-0

**Published:** 2015-08-23

**Authors:** Nan Li, Chen Chen, Bin Wang, Shaojie Li, Chaohe Yang, Xiaobo Chen

**Affiliations:** State Key Laboratory of Heavy Oil Processing, China University of Petroleum, Qingdao, 266580 China

**Keywords:** Shale oil fluid catalytic cracking, Nitrogen compounds and condensed aromatics, Structure characterization

## Abstract

Untreated shale oil, shale oil treated with HCl aqueous solution and shale oil treated with HCl and furfural were used to do comparative experiments in fixed bed reactors. Nitrogen compounds and condensed aromatics extracted by HCl and furfural were characterized by electrospray ionization Fourier transform cyclotron resonance mass spectrometry and gas chromatography and mass spectrometry, respectively. Compared with untreated shale oil, the conversion and yield of liquid products increased considerably after removing basic nitrogen compounds by HCl extraction. Furthermore, after removing nitrogen compounds and condensed aromatics by both HCl and furfural, the conversion and yield of liquid products further increased. In addition, N_1_ class species are predominant in both basic and non-basic nitrogen compounds, and they are probably indole, carbazole, cycloalkyl-carbazole, pyridine and cycloalkyl-pyridine. As for the condensed aromatics, most of them possess aromatic rings with two to three rings and zero to four carbon atom.

## Introduction

Since the storage of conventional crude oil has decreased considerably in recent years. The unconventional gas and oil resources, such as oil sands bitumen, extra-heavy oil and oil shale have attracted more and more attention in the world [1]. It has been conservatively estimated that the reserve of world oil shale resources is about 8.0 trillion barrels, while the total known conventional oil resources have a lower reserve of 2.4 trillion barrels [2–4]. Oil shale has been regarded as an important substitutable fuel resource since its huge reserve. Through the process of retorting, the oil shale can be converted into fuel feedstock called shale oil. However, the shale oils contain a considerable amount of nitrogen compounds, condensed aromatics and unsaturated hydrocarbons, which may cause many problems during the transportation, storage and processing [5].

As crude oil supply all over the world is becoming heavier and inferior in quality and the demand for middle distillate is larger, fluid catalytic cracking (FCC) process is still the key process in oil refinery and is now in rapid development [6]. The retardation effects of nitrogen compounds and condensed aromatics in FCC process have to be figured out if shale oil is used to produce clean fuel. The retarding effects of basic nitrogen compounds may be explained by two mechanisms [7–9]. First, the basic nitrogen compounds reversibly absorb on the Brønsted or Lewis acid centers and then poison the acid centers, thus weaken other hydrocarbons cracking ability. Second, since their big size and aromatic nature, the basic nitrogen compounds may be the precursors of coke. Li et al. [10] found that the retarding effects of non-basic compounds and condensed aromatics in catalytic cracking of coker gas oil are not as serious as those of basic nitrogen compounds.

So it is very meaningful to characterize the nitrogen compounds and the condensed aromatics in shale oil and figure out their influence on FCC process. The Fourier transform ion cyclotron resonance mass spectrometry (FT-ICR MS) has largely been used to characterize petroleum at the molecular level [11]. Since electrospray ionization (ESI) has the advantage of easy to operate and high selectivity for polar species, it has been used in characterizing polar compounds in unconventional oil, petroleum distillates and crude oil fractions [12–15]. Chen et al. [16] have characterized the nitrogen compounds in hydro-treated and untreated shale oil by ESI FT-ICR MS and reported that the N_1_ species in Fushun shale oil are probably pyridine, indole, carbazole, benzocarbazole and their derivatives. However, besides nitrogen compounds, there are a large amount of condensed aromatics in shale oil. Gas chromatography and mass spectrometry (GC–MS) is often used to characterize condensed aromatics in oil samples since they cannot be ionized [17]. To some extent, there is still little detail information about the condensed aromatics and nitrogen compounds in shale oil. And the kinds of nitrogen compounds and condensed aromatics that are easily removed by acid–base neutralization and solvent extraction need to be further figured out.

In this article, basic nitrogen compounds, non-basic nitrogen compounds and condensed aromatics were selectively separated by acid–base neutralization and solvent extraction, respectively. The retardation effect of nitrogen compounds and condensed aromatics on shale oil FCC process was investigated by contrast experiments of different shale oils in a fixed bed reactor over a commercial equilibrium catalyst. And the nitrogen compounds and condensed aromatics were characterized by FT-ICR MS and GC–MS respectively.

## Experimental

### Experimental scheme

**Scheme 1:**
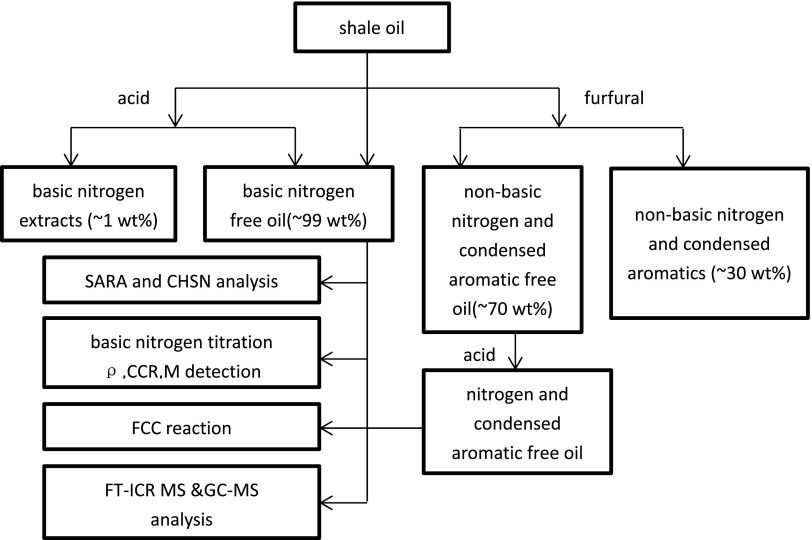
Experimental scheme

The whole procedure of the experiments is represented in Scheme 1. The basic nitrogen refers to nitrogen compounds which can be titrated by perchloric acid in acetic acid and benzene sample solutions. On the contrary, non-basic nitrogen refers to nitrogen compounds which cannot be titrated by perchloric acid. 1 mol/L HCl aqueous solution was first used as an extraction agent to remove basic nitrogen compounds from shale oil and got sample B. HCl with the same concentration and furfural were used to remove both nitrogen compounds and condensed aromatics and got sample C. The composition analysis of saturates, aromatics, resins and asphaltenes (SARA), elements content (CHSN), basic nitrogen contents, density (*ρ*), conradson carbon residue (CCR), molecular weight (*M*) and the FCC reaction performance were carried out. The SARA analysis abided by the procedure described by Liang et al. [18] and the basic nitrogen contents were detected by benzene-glacial acetic acid potentiometric titration (SH/T 0162-92 by RIPP of Sinopec). The elements contents were measured by a Vario EL III elemental analyzer and CCR was analyzed by the Chinese standard analytical method for the detection of carbon residue. Pycnometer method was used to measure density and vapor pressure osmometry was used to detect molecular weight. Moreover, negative ion ESI FT-ICR MS and GC–MS were used to characteristic the nitrogen compounds and condensed aromatics in three oil samples.

### Apparatus and catalyst of FCC reaction

**Scheme 2:**
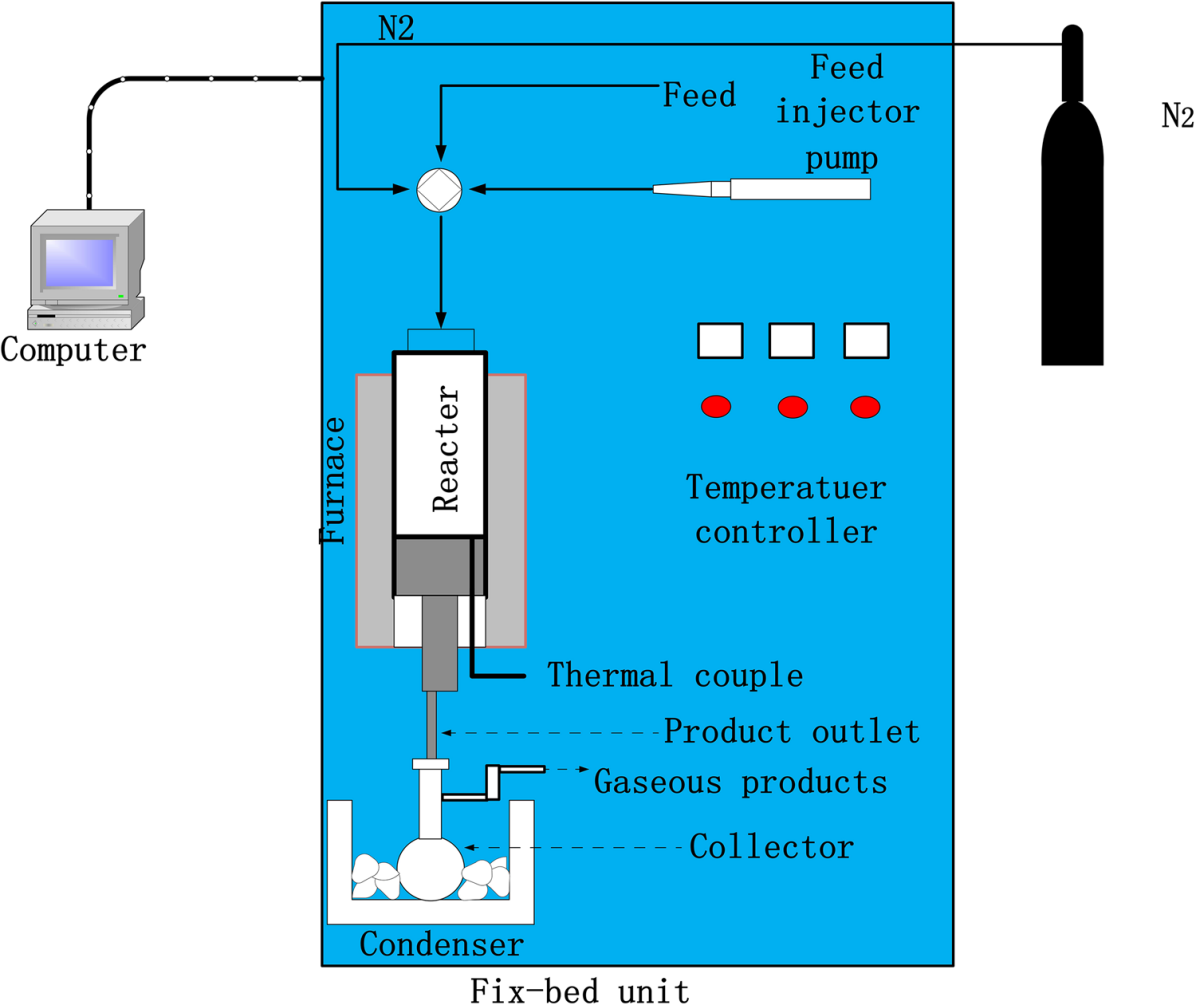
Schematic of the experimental apparatus

The FCC reactions were performed in a fixed bed reactor for heavy oil. In general, the feedstock was injected by a pump through the four-way valve which was the key part of the apparatus. Through a moveable tube the feedstock was injected into the reactor that was surrounded by a furnace to provide heat during reaction. There was a product collector under the reactor. The liquid products were collected by condensator and the gas products were collected by water gas displacing principle (Scheme 2).

The catalyst used in FCC reactions was LTB-2: MMC-2 = 1:1 (mass ratio). The LTB-2 which had already been industrialized was based on ZSM-5 zeolite to maximize propylene and it was developed by China University of Petroleum (East China). MMC-2 used ZSP zeolite as active compounds. It was developed by RIPP of Sinopec and also had been industrialized. The mainly properties of the two catalysts are listed in Table 1.

**Table 1:** Properties of equilibrium FCC catalyst

Property	LTB-2	MMC-2
*ω* (Al_2_O_3_) (%)	37.5	48.8
Microactivity index	55	73
Surface area (m^2^/g)	211	230
Pore volume (cm^3^/g)	0.26	0.29
Attrition index (*ω*) (%/h)	2.9	1.6
Particle size distribution (µm)		
0–40	5.2	15.3
0–149	96.8	92.3
Average particle size	71.0	75.2

During all the tests, the mass balances were all between 95 and 100 wt% of the feed. In this work, conversion is defined as the sum of dry gas, liquid petroleum gas, gasoline, diesel and coke; the total liquid products are referred to the sum of liquid petroleum gas, gasoline and diesel yields; the sum of the yields of gasoline and diesel is defined as the light oil.

### Products analysis

The gas products were detected by an Agilent 6890 gas chromatograph. The volume percentage of H_2_, N_2_ and C_1_–C_6_ hydrocarbons can be obtained. And according to the equation of state for ideal gases, these data can be converted into mass percentages. The collected liquid products were weighted and analyzed by stimulated distillation (ASTM-2887-D). The gasoline, diesel, heavy oil were quantified according to the temperature of initial boiling point (IBP) to 205, 205–350 and 350+ °C, respectively. The coke content on catalyst was detected by infrared carbon–sulfur determinator.

### Catalysts characterization

The total acid sites number of catalysts were characterized by ammonia temperature programmed desorption (NH_3_-TPD). About 100 mg of a sample was loaded into the apparatus, pretreated under helium flow at 600 °C for 0.5 h, brought into contact with NH_3_ after being allowed to cool to 100 °C and then heated at a rate of 10 °C/min to 800 °C.

Fourier transform infrared (FT-IR) spectra were recorded on a Nicolet 6700 spectrometer equipped with a MCT liquid nitrogen cooled detector. The spectra of the samples were recorded by accumulating 32 scans at 4 cm^−1^ resolution. The amount of pyridine absorbed on acid sites with different acid types of the catalyst was obtained from infrared transmittance spectroscopy.

### ESI FT-ICR MS analysis

10 mg of oil sample was mixed with 1 mL of toluene and then 2 μL of the solution mixture was removed diluted with 1 mL of toluene/methanol (1:1 v/v) solution. All solvents used were analytical reagent grade.

Shale oil before and after extraction and its extracts were characterized by a Bruker Apex-Ultra FT-ICR mass spectrometer equipped with a 9.4T super conducting magnet at the China University of Petroleum (Beijing, China). The sample solutions were infused via an ApolloIIelectrospray source at 150 μL/h by a syringe pump. Typical conditions for positive ion (or negative ion) formation were the following: emitter voltage, −4.0 kV (or 3.5 kV); capillary column introduction voltage, −4.5 kV (or 3.0 kV); capillary column end voltage, −320 V (or 320 V). Ions were accumulated for 0.1 s in a hexapole with 2.4 V (or −2.4 V) of direct current voltages and 300 V (peak-to-peak) radio frequency (rf) amplitudes. The optimized mass for quadrupole 1 (Q1) was at *m/z* 250 to get a broad range. And octopoles were operated at 5 MHz at a peak-to-peak rf amplitude of 400 V, in which ions accumulated for 1 s. The flight time of ions to analyze pool was 1.3 ms. ICR was operated at a 11.75 db attenuation, 150–750 Da mass range, 4 M acquired data size. The time-domain data sets were co-added for 64 acquisitions. The analytical parameters and instrument calibration for the data obtained by the ESI FT-ICR mass spectrometer are in accordance with the research of Shi et al. [19] and Zhu et al. [20].

### GC–MS analysis

A Thermo-Finnigan Trace DSQ GC–MS coupled with a HP-5MS column (30 m × 0.25 mm × 0.25 μm) was used to analyze the composition of condensed aromatics in the extract fraction of shale oil. The GC oven was maintained at 35 °C for 1 min, increased to 300 at 2 °C/min and then kept at 300 °C for 10 min. The sample was injected at 300 °C. The electron impact (EI) ionization source was operated under 12 eV ionization energy. The mass range was set to 35–500 Da at a 1 s scanning period. The ion source temperature was 200 °C, and the ion current was 250 μA.

## Results and discussions

### The effect of extraction on shale oil

**Fig. 1:**
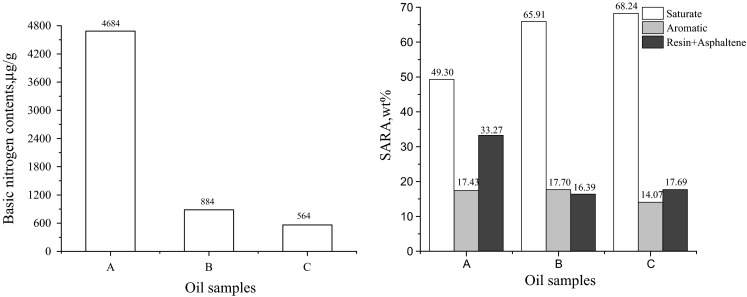
Basic nitrogen contents and SARA compositions of oil samples. *A* Untreated shale oil, *B* raffinate oil extracted by HCl, *C* furfural raffinate oil further extracted by HCl

The basic properties, basic nitrogen contents and SARA compositions of both shale oil extracted by HCl (sample B) and furfural raffinate oil further extracted by HCl (sample C) varied sensibly compared with those of untreated shale oil, as shown in Table 2 and Fig. 1. After extraction, the oil samples became superior, as the molecular weight, density and Conradson carbon residue all decreased considerably. And the nitrogen contents of both sample B and C decrease by more than 50 %, which can be explained by the removal of basic nitrogen contents. As shown in Fig. 1, after extraction by HCl, more than 80 % basic nitrogen compounds were removed. On the contrast, although sample C was first extracted by furfural and then extracted by HCl, the contents of basic nitrogen did not decrease sharply, only about 88 % nitrogen was removed. As for the SARA compositions, there were significant differences between sample A and sample B, but only slight changes were observed between sample B and sample C. After extraction by HCl, saturate as well as resin and asphaltene changed considerably, with a sharp increase of 16.61 % and a great decrease of 16.88 %, respectively, while the aromatic increased a little. This kind of change may be caused by inevitable remove of the heavy components in shale oil during the HCl extraction, thus the relative components of resin and asphaltene decrease, while the relative components of saturate and aromatic increased. On the contrary, the SARA composition of sample C only showed a slight increase in saturate (2.33 wt%) and a slight decrease in aromatic (3.63 wt%) compared with those of sample B. As for the resin and asphaltene components, the percentage of weight almost remained the same, which means that furfural mainly gathered the aromatic hydrocarbons.

**Table 2:** Properties of three oil samples

Oil samples	*M*	*ρ*_20 °C_ (kg/m^3^)	CCR (wt%)	Elemental analysis (wt%)			
				C	H	N	S
Sample A	347.4	908.2	2.25	84.82	12.12	1.22	0.41
Sample B	277.9	888.2	1.33	84.84	12.67	0.63	0.33
Sample C	284.5	874.9	0.99	84.84	13.18	0.41	–

A, untreated shale oil; B, raffinate oil extracted by HCl; C, furfural raffinate oil further extracted by HCl. *M*, molecular weight; *ρ*_20 °C_, density at the temperature of 20 °C; CCR, conradson carbon residue

### FCC performance of shale oil before and after extraction

**Fig. 2:**
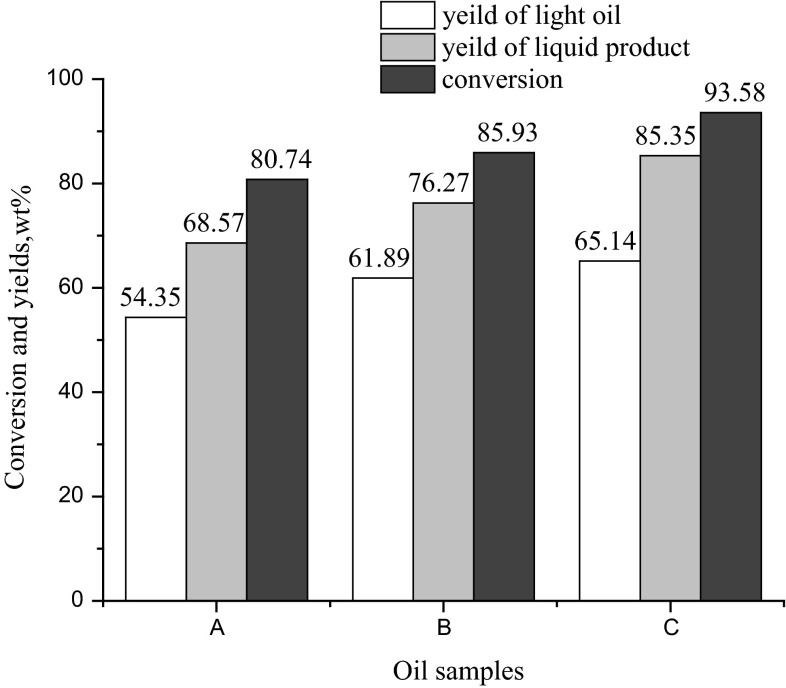
Conversion and yield for oil samples in FCC reactions. *A* Untreated shale oil, *B* raffinate oil extracted by HCl, *C* furfural extracted oil further extracted by HCl

Untreated shale oil, raffinate oil extracted by HCl and furfural raffinate oil further extracted by HCl were used to conduct comparative tests in fixed bed reactor under the following conditions: reaction temperature of 520 °C, catalyst-to-oil (CTO) ratio of 6.0, and weight hourly space velocity (WHSV) of 12 h^−1^. The conversion, yields of liquid products and yields of light oil are presented in Fig. 2. Under the same reaction conditions, the FCC performances of shale oil extracted by HCl are superior to shale oil without any treatment, and shale oil extracted by both furfural and HCl showed the best results. Comparing sample A with B, after removing basic nitrogen the conversion, yields of liquid products and yields of light oil increased from 80.74, 68.57 and 54.35 to 85.93, 76.27 and 61.89 %, respectively. Comparing sample B with C, further removing condensed aromatics led to an increase of 7.65 % in conversion, 9.08 % in yield of liquid products and 3.25 % in yield of light oil. This result suggests that the non-basic nitrogen compounds and condensed aromatic also influence FCC performances considerably which is different from what Li et al. [10] have found. This may because the total percentage of aromatic, resin and asphaltene in shale oil was up to 50 %, which was much higher than the percentage in CGO. Without extraction by furfural, the percentage of condensed aromatics in shale oil is high. Through strong adsorption and high dehydrogenation, non-basic nitrogen compounds and condensed aromatics became coke precursor that would cover acid centers and therefore limit the catalytic cracking process, resulting in a decrease in conversion as well as yield of light oil and liquid products.

**Fig. 3:**
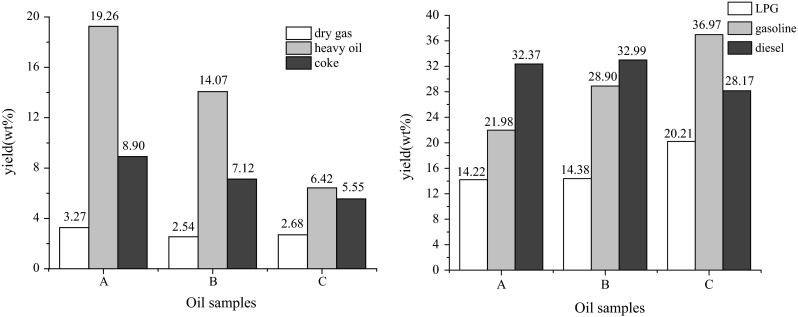
Product distribution for oil samples in FCC reactions before and after treatment. *A* Untreated shale oil, *B* raffinate oil extracted by HCl, *C* furfural extracted oil further extracted by HCl

As for the product distribution shown in Fig. 3, shale oil after furfural and HCl extraction showed a more desirable product distribution than untreated shale oil. Extraction with HCl resulted in a decrease in the yield of dry gas, heavy oil and coke and an increase in the yield of LPG and gasoline. The yield of heavy oil decreased from 19.26 to 14.07 % after removing the basic nitrogen compounds, and then dramatically reduced to 6.42 % after removing both basic nitrogen, non-basic nitrogen and condensed aromatic compounds. This is due to the removal of basic nitrogen compounds which can poison acid centers and non-basic nitrogen as well as condensed aromatic compounds which readily evolve toward coke and then cover the acid sites through extraction treatment.

As can be seen from Fig. 3 after removing the basic nitrogen compounds, the yield of LPG did not change sensibly but the yield of gasoline increased dramatically. And when both the basic nitrogen and non-basic nitrogen, condensed aromatics were removed, the yields of LPG and gasoline increased from 14.38 and 28.90 to 20.21 and 36.97 %, respectively. In addition, the yield of diesel kept constant and even decreased after extraction treatment. This may because the competitive adsorption effect between nitrogen compounds, aromatic compounds, crackable components and themselves. Acid centers on catalysts must be provided for cracking during FCC process. Nitrogen compounds in shale oil are a key factor in competitive adsorption effect since they can strongly adsorb on the acid centers and hardly crack. Condensed aromatics have the ability of strong adsorption and dehydrogenation, which may form coke and therefore block the pore openings. Thus, the acid centers for crackable compounds are less since the existence of competitive adsorption effect. Once the strong adsorptive compounds are removed, the reactions of cracking compounds are no longer restricted. The oil sample using both furfural and HCl extracted contains the least amount of strong adsorptive compounds. So it has more acid sites for diesel further crack into gasoline. And the yield of coke decreased from 8.90 to 7.12 % after HCl extraction and then decreased to 5.55 % after furfural and HCl extraction. This is due to the different content of nitrogen compounds and condensed aromatics, which adsorb on acid centers and evolve toward coke in each oil sample.

### The acidity of different coked catalysts

There are two types of acidic sites on a cracking catalyst [21–23]. Brønsted acid sites are capable of donating a proton to a base, while Lewis acids can accept unpaired electrons from a base. Identification of the acid type and number of both regenerated and spent catalysts helps to investigate the influence of components in shale oil on catalyst acidity. The results are shown in Table 3. As observed, the coked catalyst which used untreated shale oil as feedstock lost nearly 65 % acid sites contents. And the loss ratio of catalyst of sample B and C was 56.2 and 59.9 %, respectively. The loss of Lewis acid was larger than that of Brønsted acid. After HCl extraction, the loss rate of acid sites reduced considerably. But after further extraction by furfural, the loss rate of acid sites did not change so much. We can infer from that, unlike the nitrogen compounds absorbing on the acid sites, the condensed aromatic may just block on the surface on catalyst and dehydrogenate to form coke precursor.

**Table 3:** Changes in acid sites content of coked and equilibrium catalysts

Catalyst		*L*	*B*	Total acid sites content
Cat. E	Initial acid sites content (μmol/L)	146.5	15.5	162
Cat. A	Residual acid sites content (μmol/L)	47.3	8.7	56
	Loss rate (%)	67.7	43.9	65.4
Cat. B	Residual acid sites content (μmol/L)	62.5	8.5	71
	Loss rate (%)	57.3	45.2	56.2
Cat. C	Residual acid sites content (μmol/L)	57.7	7.3	65
	Loss rate (%)	60.6	52.9	59.9

Loss rate = (initial acid sites content − residual acid sites content)/initial acid sites content

Cat. E, catalyst before reaction; Cat. A, catalyst after reaction with shale oil; Cat. B, catalyst after reaction with HCl extracted shale oil; Cat. C, catalyst after reaction with HCl and furfural extracted shale oil

## ESI FT-ICR MS analysis of nitrogen compounds in shale oil

**Fig. 4:**
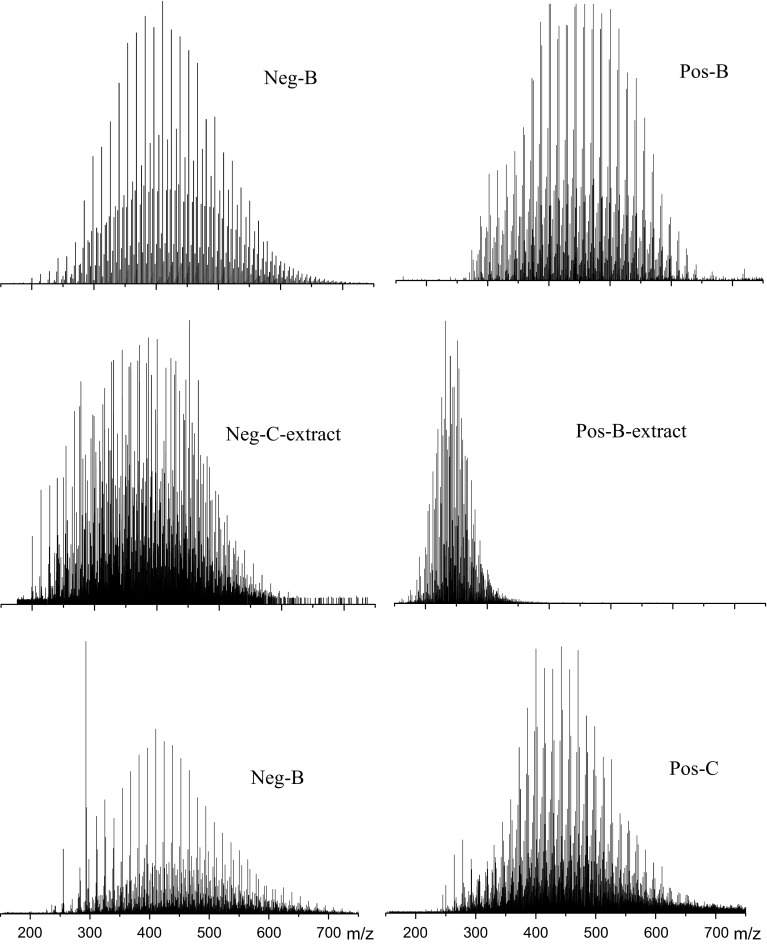
Broadband negative and positive ion ESI FT-ICR mass spectra of oil samples

The details about structures of nitrogen compounds in raffinate oils extracted by HCl (sample B), extract phrase of HCl (sample B-extract), extract phrase of furfural (sample C-extract) and furfural raffinate oil further extracted by HCl (sample C) were characterized by positive and negative ion ESI FT-ICR mass spectra. The positive ion mode can detect basic nitrogen compounds while negative ion mode can detect non-basic nitrogen compounds. As showed in Fig. 4, the molecular weight of non-basic nitrogen compounds ranges from *m/z* 200 to *m/z* 700. The mass spectra of sample B, which is the shale oil after removing most basic nitrogen compounds, center at about *m/z* 410. The mass spectra of sample C-extract, which is mostly the non-basic nitrogen compounds and condensed aromatics, center at *m/z* 360. But its shape is not a standard normal distribution. The mass spectra of sample C, which is shale oil after removing both nitrogen compounds and condensed aromatic, center at *m/z* 420. As for the mass distribution of basic nitrogen compounds, the sample B centers at 440 *m/z* and sample C centers at 450 *m/z*. And sample B-extract which is the basic nitrogen compounds extracted by HCl centers at 240 *m/z*. We can found that after HCl or furfural extraction, the mass distribution changes to a higher range, which manifests that both HCl and furfural tend to extract molecular with less molecular weight.

**Fig. 5:**
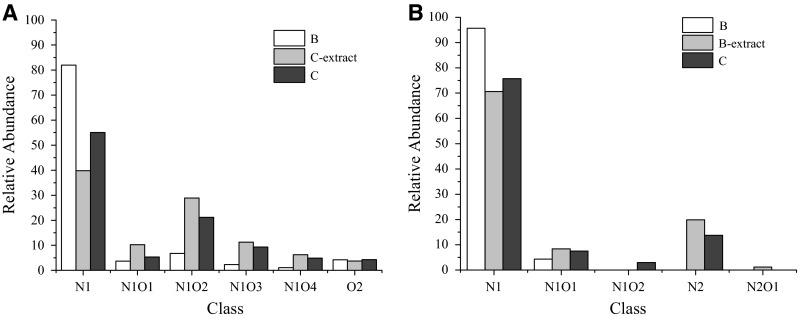
Relative abundance of heteroatom class species in oil samples. **a** class species in non-basic nitrogen compounds, **b** class species in basic nitrogen compounds

The relative abundance of different class species is defined as the magnitude of each peak divided by the sum of the magnitudes of all identified peaks (excluding the isotopic peaks) in the mass spectrum [10]. Different oil samples may have the same relative abundance distribution, but the amount of that same species may not be the same, because absolute amount of other species is different. From Fig. 5, we can see that non-basic nitrogen compounds of the oil samples contain heteroatom of N_1_, N_1_O_1_, N_1_O_2_, N_1_O_3_, N_1_O_4_ and O_2_. While the species of basic nitrogen compounds are simpler, contain heteroatom of N_1_, N_1_O_1_, N_1_O_2_, N_2_ and N_2_O_1_. And the N_1_ class species are the most abundant in all the oil samples.

**Fig. 6:**
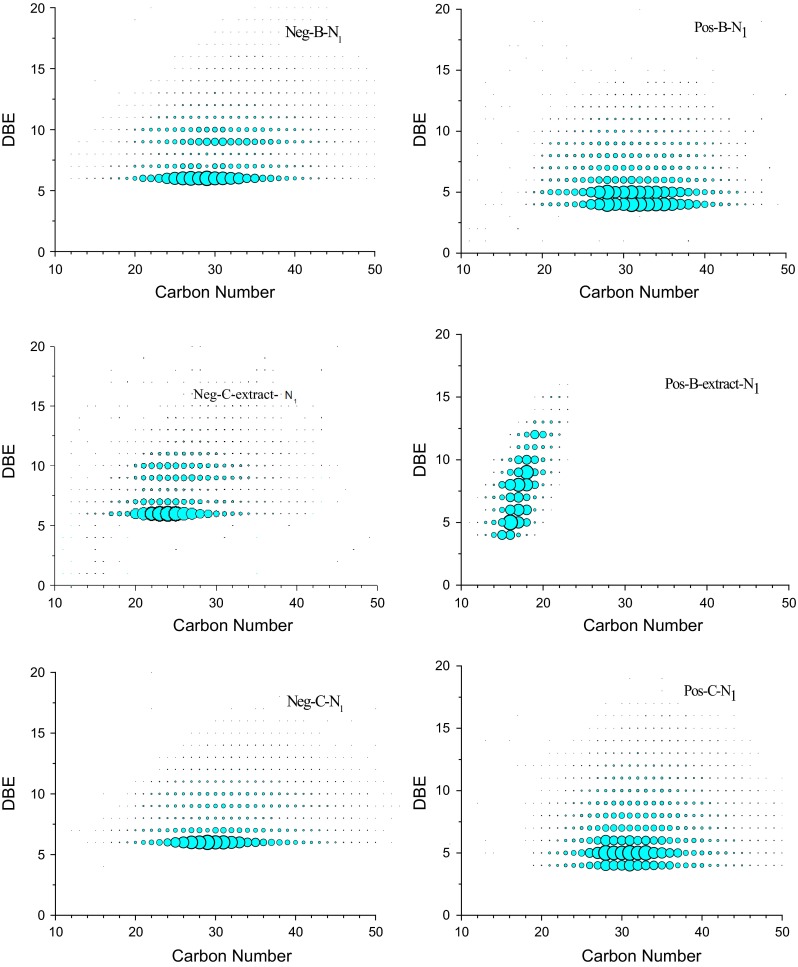
Iso-abundance plots of DBE versus carbon number of N_1_ class species in oil samples

To get more details about the nitrogen compounds in shale oil, iso-abundance dot-size-coded plots of shale oil before and after extraction and its extracts getting from the data in FT-ICR MS analysis are shown in Fig. 6. Each species on the dot-size-coded plots was distributed over a wide range of DBE values and carbon numbers, from which the possible structure of nitrogen compounds can be inferred. The DBE is abbreviation of double bond equivalents, which is used as a measure of aromaticity. For any elemental composition, C_*c*_H_*h*_N_*n*_O_O_S_S_, DBE is defined as follows [14]:

$${\text{DBE}} = {\text{rings}} + {\text{double bonds}} = c - h/2 - n/2 + 1$$

Figure 6 shows the iso-abundance plots of N_1_ species of both basic and non-basic nitrogen compounds in oil samples. The plots of non-basic N_1_ class species from negative ion ESI FT-ICR MS are shown on the left side of Fig. 6. The non-basic N_1_ class species in sample B range from 20 to 40 in carbon number and 6–12 in DBE. The N_1_ class species with DBE of 6, 9, 10 are of the highest relative abundance, which may be indole, carbazole and cycloalkyl-carbazole, respectively. The non-basic N_1_ class species in sample C-extract phrase of furfural have a broader distribution of DBE and carbon numbers. The highest relative abundance of N_1_ class species is the same as sample B, while the non-basic N_1_ class species in sample C only center at DBE of 6 with carbon number from 20 to 40, which most probably is indole with alkyl side chains.

As for the basic N_1_ class species in oil samples presented on the right of Fig. 6, the distribution of DBE and carbon numbers is wider than those of non-basic N1 class species. The basic N_1_ class species in sample B range from 20 to 40 in carbon numbers and 4–10 in DBE. The N_1_ class species with DBE of 4 and 5 are of the highest relative abundance, which can be inferred as pyridine, cycloalkyl-pyridine and their derivatives. The basic N_1_ class species in sample B-extract have a broader distribution of DBE with small carbon numbers. This result is consistent with the broadband mass spectra in Fig. 4, indicating that the compounds with small molecular weight are easier to extract by HCl. The basic N_1_ class species in extraction phrase of HCl range from 4 to 15 in DBE but only 12–22 in carbon numbers. The N_1_ class species in sample B-extract probably include pyridine, cycloalkyl-pyridine, dihydro-quinoline, quinolone, cycloalkyl-quinoline, acridine and their derivatives. The DBE and carbon numbers distribution of basic N_1_ class species sample C is similar to that of sample B, ranging from 4 to 15in DBE and 26–40 in carbon numbers. The compounds of the highest relative abundance are supposed to be pyridine, cycloalkyl-pyridine and dihydro-quinoline.

### GC–MS analysis of condensed aromatics

**Fig. 7:**
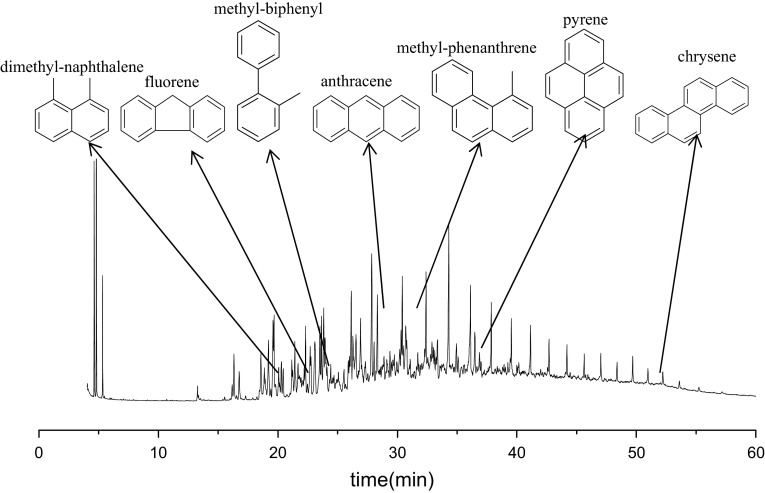
Total ion chromatogram of condensed aromatics in shale oil

Since the nonpolar condensed hydrocarbons cannot be ionized by ESI and they also influence the shale oil FCC performance, GC–MS techniques are used to characterize aromatics of furfural extraction in shale oil. The temperature of chromatographic column, it can reach, is lower than the vaporization temperature of heavy components in shale oil, so the aromatics separating from SARA method were chosen. The total ion chromatogram is shown in Fig. 7 and some of the molecular structures are drawn beyond the figure. The predominant molecular structures of the condensed aromatics consist of two to three aromatic rings with zero to four carbon atom. And some of them such as biphenyl and fluorene can crack during FCC reaction since benzene rings are only linked by C–C bonds. While others like pyrene and chrysene cannot crack because two or more benzene are bonded together. They are more likely to absorb on the surface of catalyst and jam the catalytic pores. So other crackable compounds cannot interact with the acid centers thus influence the product distribution of catalytic cracking process.

## Conclusion

Compared with untreated shale oil, more than 80 % of basic nitrogen compounds were removed after HCl extraction. But furfural raffinate oil further extracted by HCl did not show much change in basic nitrogen contents and SARA compositions. Compared with untreated shale oil, after removing basic nitrogen compounds, the FCC performance of sample B improved considerably. Moreover, after removing both nitrogen compounds and condensed aromatics, the conversion and yields of liquid products of sample C further increased. But the acid site centers on the surface of catalysts after reacting with sample B and C were almost the same. So the retardant factors during shale oil FCC processing may be described as the poison of basic nitrogen on acid centers and the strong adsorption and dehydrogenation of non-basic nitrogen compounds and condensed aromatics.

ESI FT-ICR MS analysis showed that non-basic nitrogen compounds in shale oil included N_1_, N_1_O_1_, N_1_O_2_, N_1_O_3_, N_1_O_4_ and O_2_ species. And the basic nitrogen compounds in shale oil included N_1_, N_1_O_1_, N_1_O_2_, N_2_ and N_2_O_1_ species. N_1_ class species were predominant in both non-basic and basic nitrogen compounds. And the probable structures of N_1_ class species are indole, carbazole, cycloalkyl-carbazole pyridine and cycloalkyl-pyridine. GC–MS analysis manifested that most of condensed aromatics contained two to three rings of aromatic rings with zero to four carbon atom.
